# A pro-oxidant property of vitamin C to overcome the burden of latent *Mycobacterium tuberculosis* infection: A cross-talk review with Fenton reaction

**DOI:** 10.3389/fcimb.2023.1152269

**Published:** 2023-04-19

**Authors:** Pratikkumar Gaglani, Manish Dwivedi, Tarun Kumar Upadhyay, Radhey Shyam Kaushal, Irfan Ahmad, Mohd Saeed

**Affiliations:** ^1^ Department of Life Sciences, Parul Institute of Applied Sciences and Biophysics and Structural Biology Laboratory, Center of Research for Development, Parul University, Vadodara, Gujarat, India; ^2^ Amity Institute of Biotechnology, Amity University, Lucknow, Uttar Pradesh, India; ^3^ Department of Life Sciences, Parul Institute of Applied Sciences and Animal Cell Culture and Immunobiochemistry Lab, Center of Research for Development, Parul University, Vadodara, Gujarat, India; ^4^ Department of Clinical Laboratory Sciences, College of Applied Medical Sciences, King Khalid University, Abha, Saudi Arabia; ^5^ Department of Biology, College of Sciences, University of Hail, Hail, Saudi Arabia

**Keywords:** *Mycobacterium tuberculosis*, oxidative burst, drug resistance, vitamin C, pro-oxidant property, Fenton reaction, reactive oxygen species (ROS)

## Abstract

Tuberculosis (TB), caused by the bacillus *M. tuberculosis*, is one of the deadliest infectious illnesses of our day, along with HIV and malaria.Chemotherapy, the cornerstone of TB control efforts, is jeopardized by the advent of *M. tuberculosis* strains resistant to many, if not all, of the existing medications.Isoniazid (INH), rifampicin (RIF), pyrazinamide, and ethambutol are used to treat drug-susceptible TB for two months, followed by four months of INH and RIF, but chemotherapy with potentially harmful side effects is sometimes needed to treat multidrug-resistant (MDR) TB for up to two years. Chemotherapy might be greatly shortened by drugs that kill *M. tuberculosis* more quickly while simultaneously limiting the emergence of drug resistance.Regardless of their intended target, bactericidal medicines commonly kill pathogenic bacteria (gram-negative and gram-positive) by producing hydroxyl radicals *via* the Fenton reaction.Researchers have concentrated on vitamins with bactericidal properties to address the rising cases globally and have discovered that these vitamins are effective when given along with first-line drugs. The presence of elevated iron content, reactive oxygen species (ROS) generation, and DNA damage all contributed to VC’s sterilizing action on *M. tb in vitro*. Moreover, it has a pleiotropic effect on a variety of biological processes such as detoxification, protein folding – chaperons, cell wall processes, information pathways, regulatory, virulence, metabolism etc.In this review report, the authors extensively discussed the effects of VC on *M. tb.*, such as the generation of free radicals and bactericidal mechanisms with existing treatments, and their further drug development based on ROS production.

## 1 Introduction

**Figure 1:**
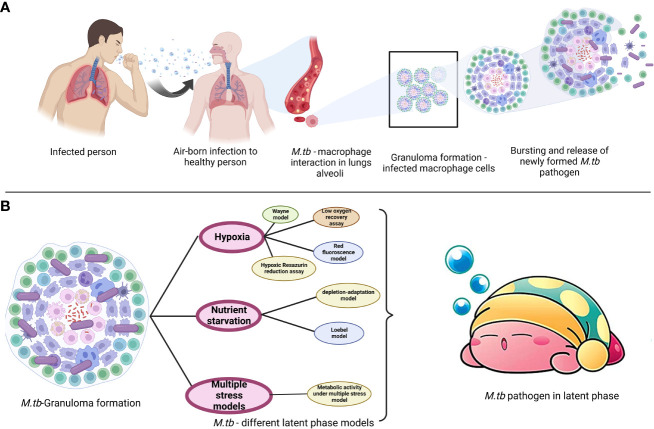
**(A)** Overview of *M. tb.* pathogenesis and **(B)** modes of development of resistivity models.

*M. tb.* a microorganism that causes TB, poses a serious threat to the public’s health. Dr. Robert Koch found the bacteria on April 3, 1882, and later gave it the name *Mycobacterium tuberculosis* (Natarajan et al., 2020). The disease is transferred through coughing when a person with TB sneezes into the air, as depicted in **Figure 1**. Although the lungs are often afflicted, pulmonary tuberculosis can potentially affect other organs (extrapulmonary TB). Men are more prone than women to get the illness, with about 90% of men developing it as adults. According to the World Health Organization (2020c), 1.4 million TB patients died in 2019 and 10 million new cases were reported each year (https://www.who.int/publications-detail-redirect/9789240013131). The COVID-19 pandemic was predicted to increase the percentage of TB deaths in 2020, and to raise the worldwide disease burden by 20% during the next five years (Alene et al., 2020 **;** Glaziou, 2020 **;** Hogan et al., 2020). Four antibiotics—isoniazid, rifampicin, ethambutol, and pyrazinamide are typically used daily for two months to treat drug-susceptible TB, followed by two antibiotics—isoniazid and rifampicin—for an additional four months. Another trend is the rise of patients with drug-resistant TB who requires second-line therapies and more intensive care. WHO reported that (https://www.who.int/publications-detail-redirect/9789240013131), 4.6% of all TB cases in 2019 were multidrug- and rifampicin-resistant. The pathogen’s intricate hydrophobic cell barrier, one of its most crucial defensive mechanisms, prevents many therapies from entering the body (Sarathy et al., 2012). Medicines that may reach their targets may lose their effectiveness as a result of target-altering mutations (e.g., isoniazid resistance caused by katG mutations; Vilchèze and Jacobs, 2014) or mutations that impede prodrug activation (e.g., rifampicin resistance caused by rpoB mutations; Zaw et al., 2018).

The death rate from TB is relatively high in the absence of treatment. Prior to the invention of drug treatments, studies and observations of the TB disease without medication revealed that approximately 70% of those with positive sputum smear, pulmonary TB, and 20% of those with positive culture (but negative smear), pulmonary TB, faced death within 10 years of their diagnosis, respectively (Tiemersma et al., 2011).

In the 1940s, the first effective pharmacological therapies were produced. The most recent WHO guidelines (https://www.who.int/publications-detail-redirect/9789240007048) recommend a 6-month regimen of ethambutol (EMB), rifampicin (RIF), pyrazinamide (PZA), and isoniazid (INH): all four medications for the first two months, followed by RIF and INH for the remaining four months. They also include novel recommendations that drug-susceptible pulmonary TB in people aged 12 and adults be treated with a four-month course of rifapentine (RFP), INH, PZA, and moxifloxacin (MXF), and that non-severe TB in children and adolescents aged 3 months to 16 years be treated with a four-month course of these drugs (two months of PZA, RIF, INH sometimes also EMB, followed by two months of RIF and INH). The WHO’s 194 Member States consistently report therapy success rates of at least 85 percent for persons completing the 6-month protocol.

It is more difficult to treat RIF-resistant tuberculosis (RR-TB) and multidrug-resistant tuberculosis (MDR-TB), which is defined by resistance to both INH and RIF (10). RR-TB treatment success rates normally range from 50% to 75% nationally, while the worldwide average has recently increased and reached 60% in the most current patient group for which statistics are available. Therapy for extreme drug-resistant tuberculosis (XDR-TB), which involves resistance to at least one fluoroquinolone plus bedaquiline or linezolid, is significantly more challenging and typically results in poor success rates.

Chemotherapy may be significantly reduced if there were drugs that eliminated TB more quickly. Bactericidal medications frequently kill *Escherichia coli* cells by causing the Fenton reaction, which produces very reactive hydroxyl radicals. Vitamin C, a compound that is known to cause the Fenton reaction, has the potential to sterilize *M. tb.* cultures, both drug-susceptible and drug-resistant cultures. While *Mycobacterium tuberculosis* is very vulnerable to it, VC is frequently resistant to both gram-positive and gram-negative pathogens. In addition to having a pleiotropic influence on a number of biological processes, VC’s capacity to kill *M. tb.* bacteria depends on elevated ferrous ion concentrations and ROS generation (Vilchèze et al., 2013).

It has been demonstrated that nanotechnology techniques offer improved intracellular Fenton reaction-based oxidative stress and Ferroptosis treatment, as well as increased vitamin C cell transport. As a result, it is now possible to use vitamin C’s pro-oxidant properties at a concentration that is physiologically appropriate (Pal and Jana, 2022).

In this review article, we address the potential advantages of incorporating VC into a TB therapy regimen and make the case that creating medications with high oxidative bursts might be very beneficial in the fight against the disease.

## 2 Medication available for treatment of *Mycobacterium tuberculosis:* - first and second-line drug

Anti-TB drugs are presently classified into four groups: Group 1 includes first-line drugs or oral antibiotics such as INH, RIF, ethionamide (ETH), and PZA; group 2 includes second-line injectable medications such as amikacin, kanamycin, and capreomycin with streptomycin (which is recognized as first-line but is also an injectable); group 3 includes fluoroquinolones; and group 4 includes second-line bacteriostatic medications such as ethionamide/prothionamide (**Table 1**) (Caminero and Scardigli, 2015). The European Respiratory Society (ERS) and the United States National Tuberculosis Controllers Association (NTCA) have endorsed a guideline for the management of medication-sensitive tuberculosis (TB) developed jointly by the American Thoracic Society (ATS), the Infectious Disease Society of America (IDSA), and the United States Centers for Disease Control and Prevention (US-CDC) (ERS). According to this recommendation, EMB, PZA, RIF, and INH for 2 months, followed by 4 months of RIF and INH, is the optimum regimen for treating persons with TB infection by bacteria that are not drug-resistant. If medication susceptibility data is available and the isolate is susceptible to both INH and RIF, EMB may be stopped (Nahid et al., 2016).

**Table 1:** Groups of drugs, molecular structure and route of drug administration in the human body.

Drug stage	Name of anti-tuberculosis drug	Structure of drug molecule	Drug-dose administration	Reference
**First line Drugs**	Cycloserine	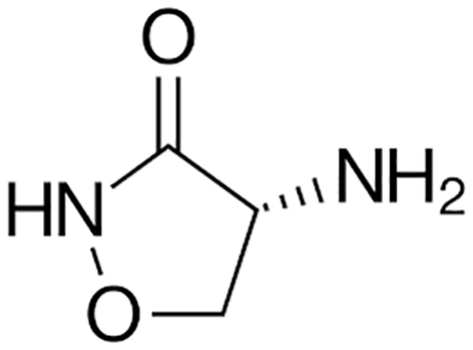	Oral	**(** Shete et al., 2019 **)**
	Isoniazid	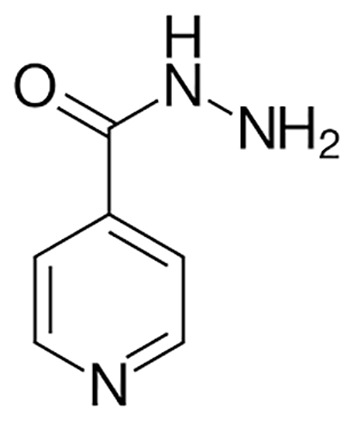	Oral or Im, Iv	
	Ethambutol	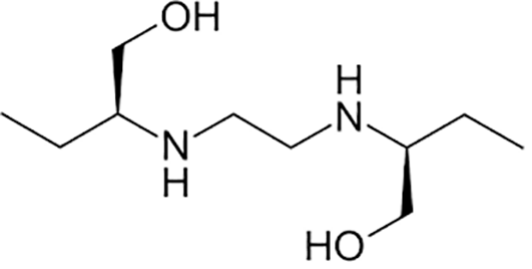	Oral	
	Rifampicin	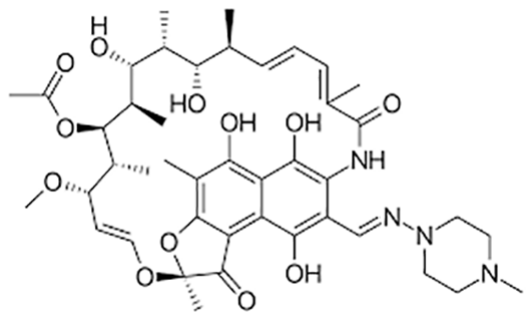	Oral or Iv	
	Pyrazinamide	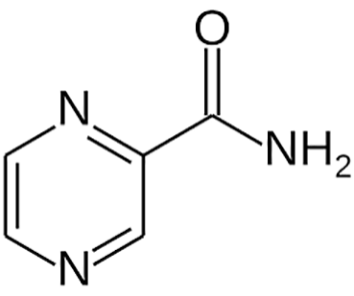	Oral	
**Second line Drugs**	Capreomycin	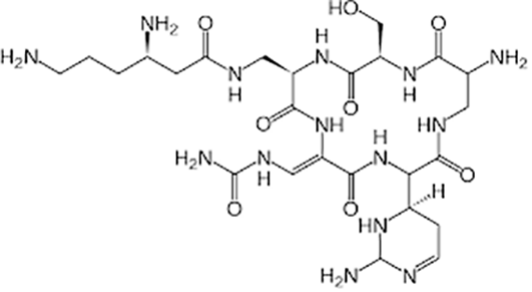	Im or Iv	
	Ethionamide	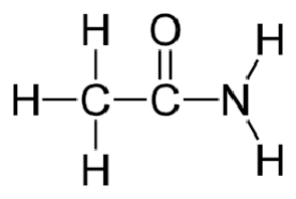	Oral	
	Amikacin/kanamycin	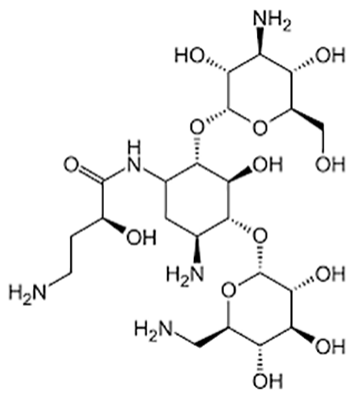	Im or Iv	
	Streptomycin	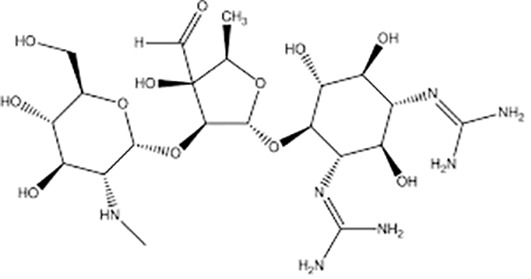	Im or Iv	
**Anti-TB drugs with less data available on safety and effectiveness**	High-dose isoniazid	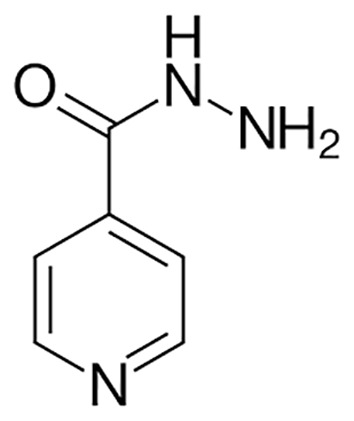	Oral or Iv	**(23)** **(** Somoskovi and Salfinger, 2014 **)**
	Para-amino salicylic acid	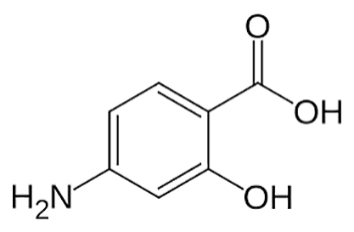	Oral or Iv	
	Clavulanate	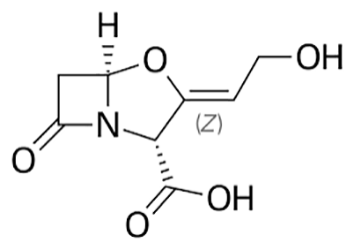	Oral or Iv	
	Levofloxacin	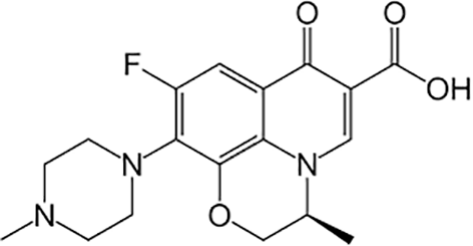	Oral or Iv	
	Imipenem–cilastatin	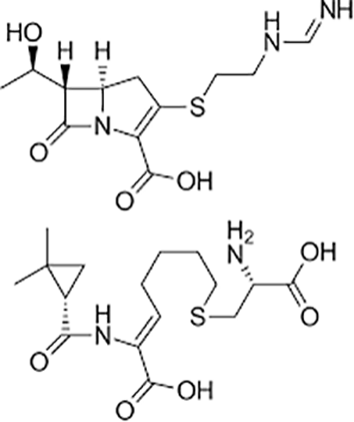	Iv	
	Moxifloxacin	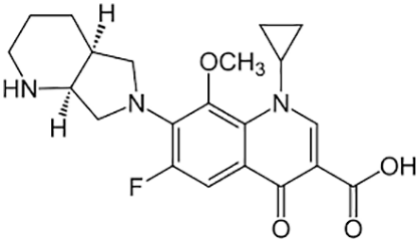	Oral or Iv	
	Meropenem	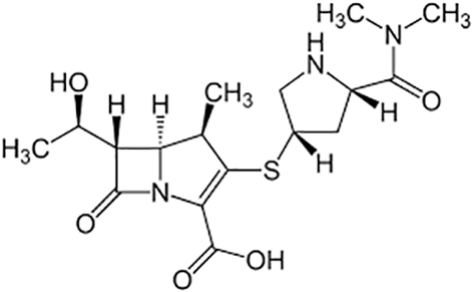	Iv	
	Bedaquiline	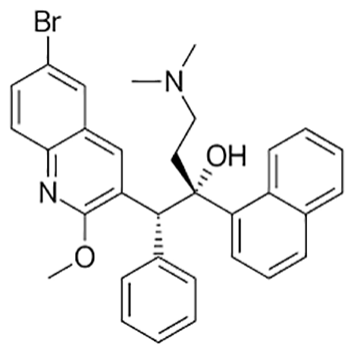	Oral	
	Delamanid	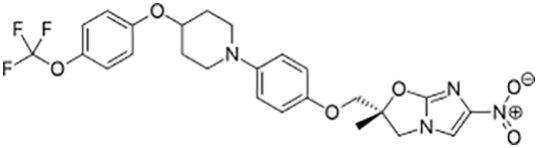	Oral	
	Linezolid	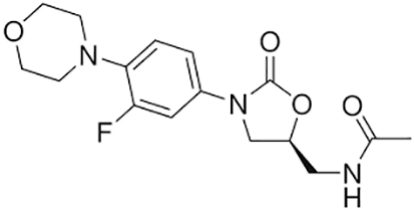	Oral or Iv	
	Clofazimine	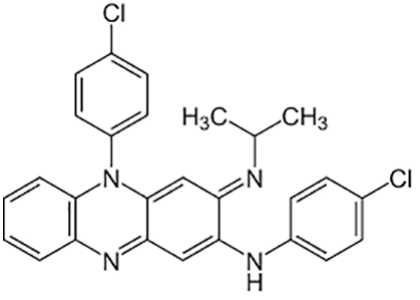	Oral	

## 3 Development of drug resistant tb

### 3.1 Restriction of access to target

#### 3.1.1 Impermeability of cell wall

The envelope of a mycobacterial cell is generally composed of three components: 1) Peptidoglycan (PG) 2) Arabinaglycan (AG) 3) Mycolic acids with long chains (Alderwick et al., 2015 **;** Daffé, 2015 **;** Jankute et al., 2015). The permeability based on the cell wall barrier protects the bacteria from harmful environments and helps the bacterium to develop resistance to a variety of medications. Hence, if these components are damaged, it may impair the function of the cell wall and increase the susceptibility of some anti-mycobacterial drugs (Jackson et al., 2013). The enzymes involved in cell wall integrity play an important role in the development of drug resistance in *Mycobacterium* cells (Jackson et al., 2013 **;** Xu et al., 2014). Peptidoglycan production is aided by the genes glmU, murA, murX, ponA1/A2, Ldt, whereas glmU, rmlC are involved in linker unit biosynthesis, accD6 and ag85 are involved in mycolic acid biosynthesis, and alr and ddl are involved in alanine metabolism. PonA1/A2 aids in cell wall formation, rmlC in arabinogalacton synthesis, and pks12 in phthiocerol dimycocerosates production (Nasiri et al., 2017). Mur A and Mur B biosynthetic enzymes are responsible for generating UDP- MurNAc, a crucial step in the biosynthesis of PG (Alderwick et al., 2015). A well-known MurA inhibitor is fosfomycin, a naturally occurring broad-spectrum antibiotic (Alderwick et al., 2015 **;** Moraes et al., 2015). It particularly inhibits MurA by forming a covalent adduct with a cysteine residue in the active site (Moraes et al., 2015). *M. tb.*, on the other hand, is naturally resistant to fosfomycin because its homologous cysteine residue is converted into aspartic acid (Alderwick et al., 2015). When the wild-type aspartate residue in the MurA active site is altered to cysteine, *M. tb.* produces an enzyme that is susceptible to fosfomycin (Castañeda-García et al., 2013).

Most often, β-lactam antibiotics inhibit the crucial D, D-transpeptidase action of normal penicillin-binding proteins (PBPs). These enzymes cross-link glycan chains by forming 43 peptide bonds between residues in the fourth and third positions of stem peptides (Gupta et al., 2010). L, D transpeptidases (Ldt) (Gupta et al., 2010 **;** Schoonmaker et al., 2014 **;** Basta et al., 2015 **;** Kieser et al., 2015), a second family of transpeptidases important for resistance to -lactam antibiotics such as amoxicillin and carbapenems, have also been found in *M. tb.* (Gupta et al., 2010 **;** Alderwick et al., 2015). *M. tb.* contains five Ldts enzymes (LdtMt1 to LdtMt5) that form the unusual 33 linkages between opposing stem peptides in PG. *M. tb.* strain that was discovered to be lacking in both ldtMt1 and ldtMt2 was shown to be more sensitive to the medicines amoxicillin and vancomycin, a glycopeptide (Schoonmaker et al., 2014). Moreover, two proteins, PonA1 and PonA2, are penicillin-binding proteins with dual roles that contribute in the production and maintenance of cell wall components (Jankute et al., 2015). The minimum inhibitory concentration (MIC) of β-lactams for ponA1 mutant *M. tb.* was the same as for wild-type *M. tb.*; however, ponA2 mutant cells were four to eight times more susceptible to β-lactams. Moreover, any alterations to the proteins and enzymes essential for maintaining the cell wall’s integrity may enhance sensitivity to particular drugs (De Gouw et al., 2014 **;** Farhat et al., 2013).

#### 3.1.2 Dormancy and latency

**Figure 2:**
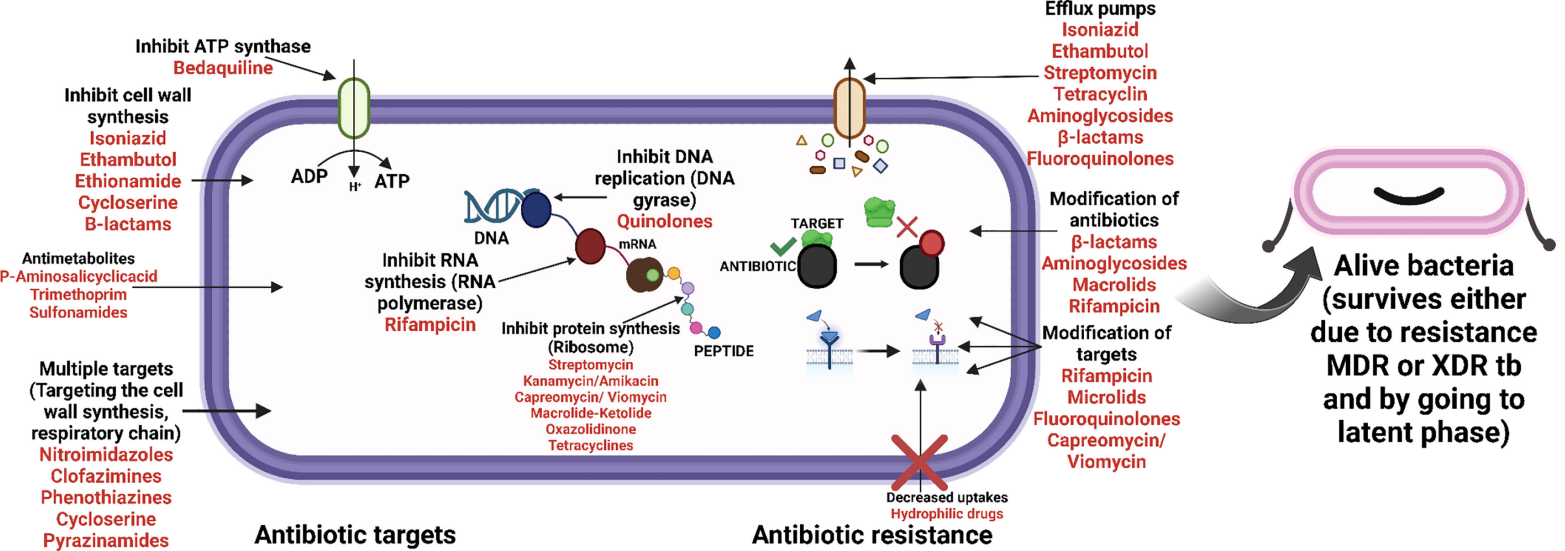
Upregulation of genes, playing vital role in helping bacteria to move to latent phase (post VC administration).

**Figure 3:**
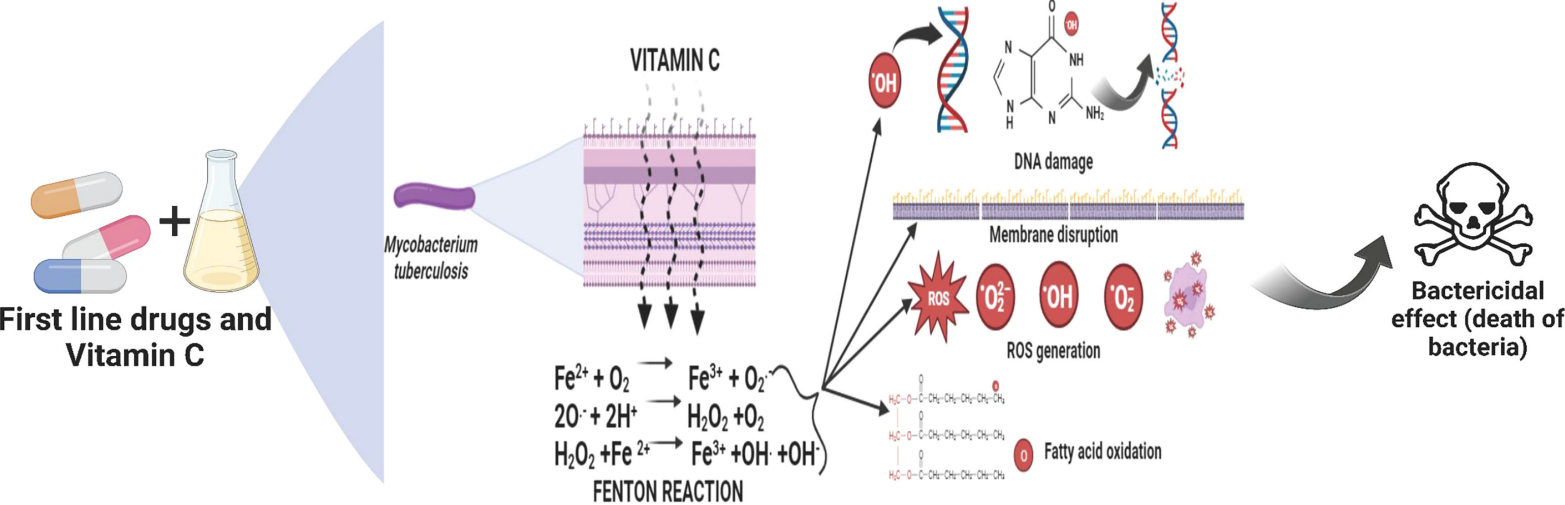
Downregulation of genes, playing vital role in helping bacteria to move into latent phase (post VC administration).

Latency refers to an infection that is asymptomatic yet still present. Dormancy is characterized by no replication and minimal or no metabolic activity. A fraction of latent cells may be required for latency and persistence even in the face of host defense and pharmacological therapy. Persisters are bacteria that survive treatment while being genetically susceptible to antibiotics (**Figures 2**, **3**) (Gomez and McKinney, 2004 **;** Chao and Rubin, 2010).

Phenotypic drug resistance is a phenomenon that occurs when *M. tb.* enters a dormant state during latent infection, which is characterized by a suspension of the majority of its metabolism and boosts the bacteria’s tolerance to antibiotics that are toxic to reproducing bacilli (Gomez and McKinney, 2004 **;** Gengenbacher and Kaufmann, 2012). Instead of chromosomal resistance mutations, this type of pharmacological tolerance is related to lower metabolic activity or decreased cell division (Garton et al., 2008). The discovery that dormant *M. tb.*, a slowly growing or non-replicating bacteria, produces antibiotic target proteins or machinery at a slower rate confirms this. Indeed, when these bacteria entered the active growth phase again, the production of antibiotic cellular targets was resumed, which led to their resensitization (Smith et al., 2013). *M. tb.* develops *in vitro* dormancy and becomes more resistant to anti-TB drugs like INH and RIF as a result of the lack of oxygen. Conversely, the anaerobic bactericidal action of metronidazole has little impact on cultures of *M. tb.* grown in aerobic settings (Wayne and Hayes, 1996 **;** Hu et al., 1998). *M. tb* starvation was another method used to encourage the transition from active to latent growth. The ability of *M. tb* to invade during the inactive sleep phase metabolically can be considered as an inherent drug-resistant process. This indicated lower rates of transcription and respiration, consumption of energy, production of fatty acids, and cell division, in addition to enhanced drug resistance to antibiotics that target active growth processes (Betts et al., 2002 **;** Wu et al., 2016).

#### 3.1.3 Efflux pumps

Mycobacterial species are naturally resistant to most medications due to the restricted permeability of their cell walls (**Figures 2**, **3**) (Brennan, 2003). In addition to increasing cell wall permeability, active efflux mechanisms also offer resistance by eliminating drug molecules that enter the cell. These efflux pumps vigorously eject numerous antibiotics from the cell and are essential for mycobacteria’s innate resistance to different medications (Nasiri et al., 2017).

### 3.2 Modification of targets

*Mycobacteria* may develop inherent resistance to various essential antibiotics as a result of a shift in target locations (Nasiri et al., 2017). For instance, the *M. tb.* gene mfpA causes quinolone resistance (Hegde et al., 2005 **;** Tao et al., 2013; Mayer and Takiff, 2020). DNA gyrase is protected from quinolone toxicity by pentapeptide repeat proteins produced by the mfpA gene. Due to electrostatic, size, and structural similarities between MfpA and B form DNA, it has been proposed that MfpA interacts with DNA gyrase through DNA mimicry (Hegde et al., 2005). Fluoroquinolones appear to be released from their target by MfpA when it binds to gyrase rather than DNA. Drug resistance might possibly originate from methylation loss (Ferber, 2005). One possibility is the development of viomycin and capreomycin resistance when methyltransferase is dormant. Its absence leads in an unmethylated, treatment-resistant ribosome because this gene makes a rRNA methyltransferase (Maus et al., 2005). GidB, a gene that methylates 16S rRNA, is also repressed, which causes low-level streptomycin resistance (Verma et al., 2014). RNA polymerase binding protein A (RbpA), which is present in *M. tb.* and *M. smegmatis*, is a putative internal mechanism *via* which mycobacterium may increase the levels of their resistance to RIF. The RNA polymerase and this protein interact, preventing RIF from adhering there (Dey et al., 2010).

### 3.3 Acquired resistance

Anti-mycobacterial drugs connect to their target sites firmly and affinitively, preventing them from performing their normal activities. Moreover, modifications to the binding site’s structure that impair an antibiotic’s ability to bind effectively may result in resistance. Mycobacterial species frequently develop drug resistance by spontaneous mutations in chromosomal genes encoding targets, in contrast to other microbes where drug resistance is typically transferred horizontally by ambulant genetic components. **Table 2** lists the mycobacterial drug targets and any known or anticipated resistance mechanisms (Nasiri et al., 2017).

**Table 2:** Anti-mycobacterial drugs and mechanisms of drug resistance.

Drug	Gene	Cellular target	Mode of action	Experimental MIC (µg/ml)	Drug resistance-mutation	References
**SQ109**	*mmpL3*	Mycolic acids	To inhibit synthesis of lipid	*Mycobacterium tuberculosis (0.5)*	–	(Protopopova et al., 2005; Tahlan et al., 2012)
**Isoniazid**	*inhA* *katG* *Ndh* *KasA* *ahpC*	Mycolic acids	To inhibit synthesis of mycolic acid	*Mycobacterium tuberculosis* *(0.02-0.1 in7H10)*	*C-15-T SNP* *Ser-315-Thr* *Arg-13-Cys Val-18-Ala* *Gly-269-Ser* *C-39-T, G-9-A SNPs*	(Woods, 2000; Somoskovi et al., 2001; Ramaswamy et al., 2003; Zhang et al., 2005; Guo et al., 2006)
**Bedaquiline (TMC207)**	*atpE*	ATP synthase	To inhibit synthesis of ATP	*Mycobacterium tuberculosis (0.25)*	*Lle-66-Met*, *Ala-63-Pro*	(Andries et al., 2005; Segala et al., 2012)
**Rifampicin**	*rpoB*	RNA polymerase	To inhibit synthesis of RNA polymerase	*M. kansasii (2)* *M. marinum Mycobacterium tuberculosis* *(1 in 7H10)*	*Ser-450-Leu*	(OFFICIAL, 1990; Heep et al., 2001; Somoskovi et al., 2001; Philley and Griffith, 2015)
**Tetracyclines and glycylcyclines**	*16S rRNA* *Gene*	30S ribosomal subunit	To inhibit the synthesis of protein	*RGM (8-16)*	–	(Brown-Elliott et al., 2012; Kasperbauer and De Groote, 2015)
**Pyrazinamide**	*rpsA* *pncA* *panD*	Fatty acid synthase-I, ribosomal protein S1	To inhibit production of energy and trans-translation	*Mycobacterium tuberculosis* *(16-50 in LJ)*	*Deletion Ala438*, *Asp-12-Ala/Asn, Leu-85-Pro* *Thr-5-Ala* *Val-138-Aal, Ala-128-Ser*	(Somoskovi et al., 2001; Shi et al., 2011; Feuerriegel et al., 2013; Zhang et al., 2013; Zhang et al., 2014)
**B-Lactams (In combination with belactamase**	*blaC* *Pbp* *ponA*	Transpeptidases	To inhibit peptidoglycan	*Mycobacterium tuberculosis (NR)* *RGM (128)*	- - -	(Wivagg et al., 2014; Fattorini et al., 1992; Hugonnet and Blanchard, 2007; Brown-Elliott et al., 2012)
**Ethambutol**	*embCAB*	Arabinosyl transferases	To inhibit synthesis of arabinogalactan	*M. marinum (5)* *Mycobacterium tuberculosis (5 in* *7H10)* *M. kansasii (5)*	*Met-306-Val/lle/Leu*	(OFFICIAL, 1990; Brown-Elliott et al., 2012; Palomino and Martin, 2014; Philley and Griffith, 2015)
**Clofazimine**	*rv1979c* *rv0678* *rv2535c*	NADH dehydrogenase	Redox cycling interference, causing membrane destabilization and reactive oxygen species production	*Mycobacterium tuberculosis (1)*	*T-1052-C SNP* *G193 deletion, C-466-T SNPs* *G-265-T SNP*	(Hartkoorn et al., 2014; Zhang et al., 2015)
**Amikacin/Kanamycin**	*Eis* *Rrs*	30S ribosomal subunit	To inhibit the synthesis of protein.	*Mycobacterium tuberculosis (5 in 7H10)* *M. kansasii (32)*	*A-1401-G SNP* *G-37-T, G-10-A* *G-14-T SNPs* *M. marinum (32)* *RGM (64)*	(OFFICIAL, 1990; Zaunbrecher et al., 2009; Georghiou et al., 2012; Philley and Griffith, 2015; Almeida da Silva et al., 2011; Kasperbauer and De Groote, 2015)
**Cycloserine**	*cycA* *Ald* *Ddl* *Alr*	L-alanine dehydrogenase, Alanine racemase, D-Alanine-D-alanine ligase, D-serine/L- and D-alanine/glycine/D-cycloserine proton symporter	To inhibit the synthesis of peptidoglycan	*Mycobacterium tuberculosis (5–10 in BACTEC)*	*Gly-122-Ser* - G-10-T SNP	(Almeida da Silva et al., 2011;Caceres et al., 1997; Garcia Pelayo et al., 2009; Chen et al., 2012; Gu et al., 2016
**Ethionamide**	*Ndh* *ethA* *inhA* *mshA*	Mycolic acids	To inhibit the synthesis of mycolic acid	*Mycobacterium tuberculosis (5 in 7H10)*	*Arg-13-Cys, Val-18-Ala* *Leu-397-Arg, Leu-328-Met* *Ile-21-Thr/Val, Ser-94-Ala* *Val-171-Gly, Aal-187-Val*	(Morlock et al., 2003; Boonaiam et al., 2010; Brossier et al., 2011)
**P-aminosalicylic acid (PAS)**	*ribD* *folC* *thyA*	Dihydrofolate synthase, Dihydrofolate reductases	To inhibit synthesis of folate	*Mycobacterium tuberculosis (2 in 7H10)*	*G-11-A SNP* *Glu-153-Aal*, *Asn-73-Ser* *Thr-202-Ala*, *Val-261-Gly*	(Zhang et al., 2013; Zhao et al., 2014; Almeida da Silva et al., 2011)
**Capreomycin/viomycin**	*Eis* *Rrs* *tylA*	30S and 50S ribosome subunits	To inhibit the synthesis of protein	*Mycobacterium tuberculosis (10 in 7H10)*	*G-37-T, C-12-T SNPs* *A-1401-G SNP* *G-223-T SNP*	(Johansen et al., 2006; Georghiou et al., 2012; Almeida da Silva et al., 2011)
**Fluoroquinolones**	*gyrB* *gyrA*	DNA gyrase	To inhibit DNA gyrase.	*Mycobacterium tuberculosis (2 in 7H10)* *M. kansasii (2)*	*Asn-533-Thr* *RGM (4)* *Ala-90-Val*, *Asp-94-Gly/Tyr*	(OFFICIAL, 1990; Kasperbauer and De Groote, 2015; Cheng et al., 2004; Brown-Elliott et al., 2012; Philley and Griffith, 2015)

NR, not required; SNP, single nucleotide polymorphism; RGM, rapidly growing mycobacteria.

"-" Information not available.

## 4 Vitamin C - mode of action on *Mycobacterium tuberculosis*

Vitamins have long been recognized as important immune-boosting nutrients. Several studies have looked into vitamins’ capacity to combat mycobacterial development. Humans are unable to synthesis VC due to a defect in the gene that codes for the enzyme gluconolactone oxidase, thus it must be ingested as a dietary supplement (Combs and McClung, 2016). A mycobacterial infection generates reactive oxygen and reactive nitrogen intermediates that VC protects the body from (Nishikimi and Yagi, 1996). It functions as a physiological antioxidant and aids in iron transfer and collagen formation. It also boosts T-cell response and stimulates leukocyte migration at the infection site **(** Field et al., 2002). Linus Pauling proposed taking 1-3 g of VC every day to avoid the common cold and flu in 1976. Many experiments have indicated that oral VC administration is critical in the prevention and treatment of tuberculosis. A deficiency of VC has also been connected to tuberculosis infection (Andosca and Foley, 1948).

Several studies have found that orally administered VC has a stronger influence in avoiding and administering *M. tb.* transmission in animals and humans (Soh et al., 2017 **;** Di Gennaro et al., 2021); others have discovered that deficiencies in nutrition and VC deficiency in TB patients are linked to a higher risk of acquiring the disease as well as catastrophic consequences (Andosca and Foley, 1948; Mishra and Sarkar, 2015; Xiong et al., 2020). Several studies have attempted, with varied degrees of success, to explain how *M. tb.* interacts with VC. Interesting results were discovered in a study examining the synergistic effects of VC when combined with rifampicin and isoniazid. Both drug-resistant strains and the wild-type H37Rv strain displayed reduced colony-forming units when grown with VC plus rifampicin at an inadequate MIC. When VC and isoniazid were tested combined, CFU in both wild type and drug-resistant bacteria were decreased. Isoniazid and VC synergy, on the other hand, had less of an effect on resistant strains as compared to H37Rv wild type (Khameneh et al., 2016).

When oxygen is available, the Fenton and Haber-Weiss process converts ferric ions to ferrous ions, which create superoxide, hydrogen peroxide, and hydroxyl radicals (Vilchèze et al., 2013 **;** Khameneh et al., 2016). These radical moieties then damage *M. tb.* DNA and lipids, preventing the organism’s development. VC is also predicted to reduce guanosine 5’ diphosphate 3’ diphosphate (ppGpp), a molecule known to be involved in the regulation of *M. tb.* growth and stress response.

In order to sterilized drug-sensitive and treatment-resistant *M. tb.* cell cultures, Vilchèze et al. claim that VC causes the Fenton reaction (Syal et al., 2017). Researchers discovered that the capacity of VC to kill *M. tb.* cells are caused by a rise in ferrous ion concentration, which results in DNA damage, ROS production, lipid changes, and redox imbalance (Volchegorskiĭ et al., 2007; Syal et al., 2017). The sterilizing effects of VC were more prominent in tuberculosis strains lacking mycothiol (Newton et al., 2011 **;** Metaferia et al., 2007). Yet, the rate of *M. tb.* cell death may be accelerated when a mycothiol inhibitor is combined with VC or another pro-oxidant agent (Newton et al., 2011; Syal et al., 2017). VC also has an impact on lipid synthesis. The scientists discovered that *M. tb.* cells treated with VC generated two free 2-hydroxylated long-chain fatty acids (Syal et al., 2017). The study’s findings were contrasted with those of Kondo e Kanai (Di Gennaro et al., 2019), who demonstrated that 2-hydroxylated fatty acids were much more harmful to mycobacteria than similar levels of saturated fatty acids. Vichèze et al. suggest that the buildup of these lipids may contribute to TB bactericidal events. The drop in phospholipid content seen in *M. tb.* treated with VC may also have an impact on the survival of the organism (Syal et al., 2017). Guanosine 50 -diphosphate 30 -diphosphate (ppGpp), a substance known to be crucial in *M. tb.* growth control and stress response, is likewise anticipated to decrease in response to VC (Sikri et al., 2018; Vynnycky et al., 2000). The VC enhances the activity of Pyrazinamide (PZA), a crucial first-line TB treatment because of its ability to sterilise non-metabolizing or steadily metabolising continuously growing bacilli that are resistant to other medications, by addressing latent bacteria and suppressing the growth of rifampin-tolerating and rifampin-resistant bacilli (Sikri et al., 2018). Yet, this investigation produced a variety of results (Taneja et al., 2010; Albeldas et al., 2018). In the same experiment, Vilchèze, Hartman, and colleagues found that high VC concentrations kill drug-resistant and drug-susceptible TB cells and prevent the spread of drug persisters. Additionally, they found that after four weeks, *M. tb.* cells became inactive when a sub-lethal dosage of VC was coupled with the first-line antitubercular drug isoniazid (INH). No mutations that could withstand therapy were found. This demonstrates that adding a sub-inhibitory dose of VC to an INH treatment may also aid to prevent the formation of mutants that are resistant to INH (Syal et al., 2017). Contrary to INH-RIF alone, VC may enhance the therapeutic effectiveness for *M. tb.* in experimental mice, increasing the rate of bacterial clearance. Another research came to this result (Vilchèze et al., 2018).

**Figure 4:**
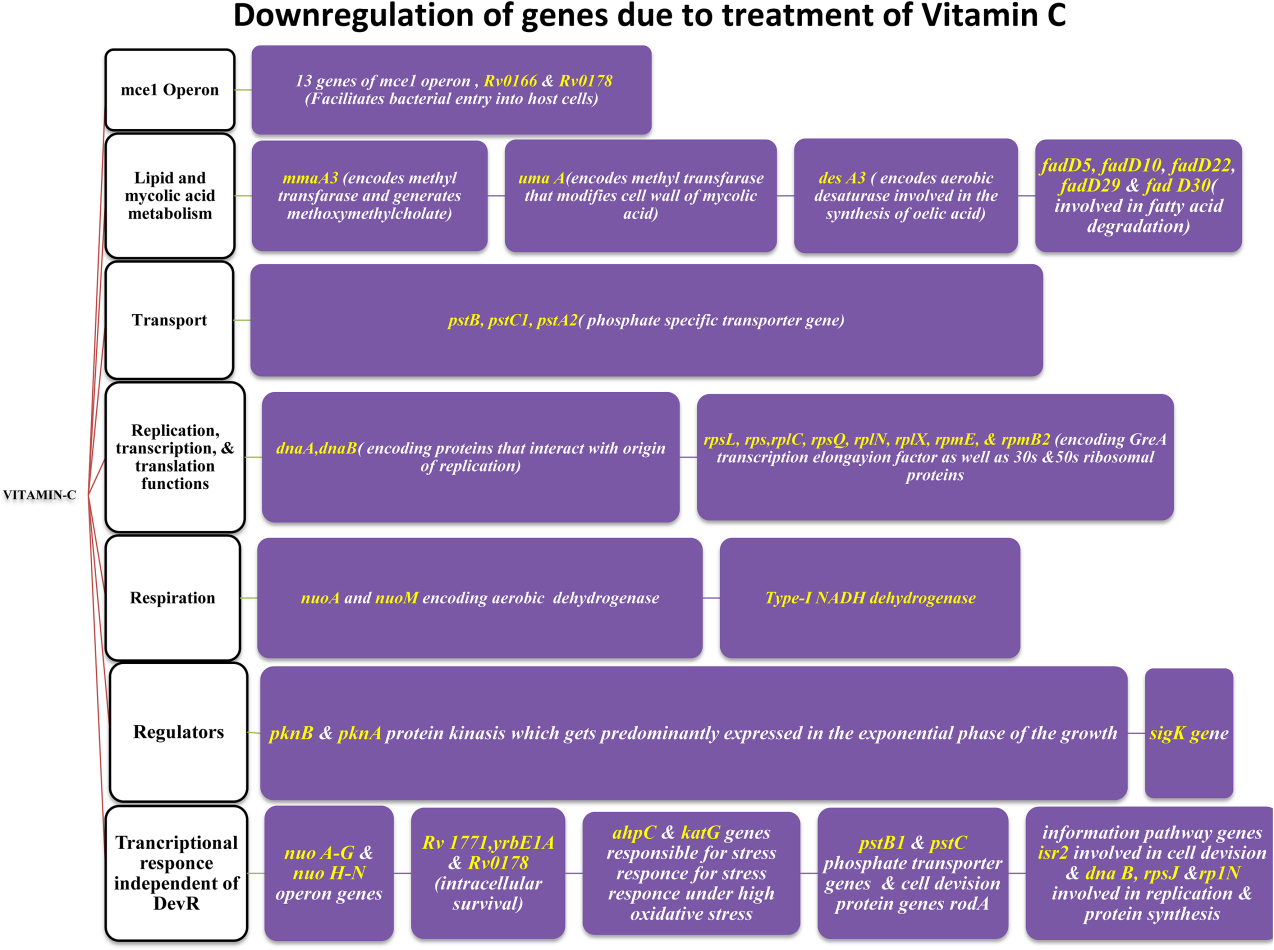
Antibiotic targeting sites (mode of action of antibiotics) and the mechanism of resistance towards them.

**Figure 5:**
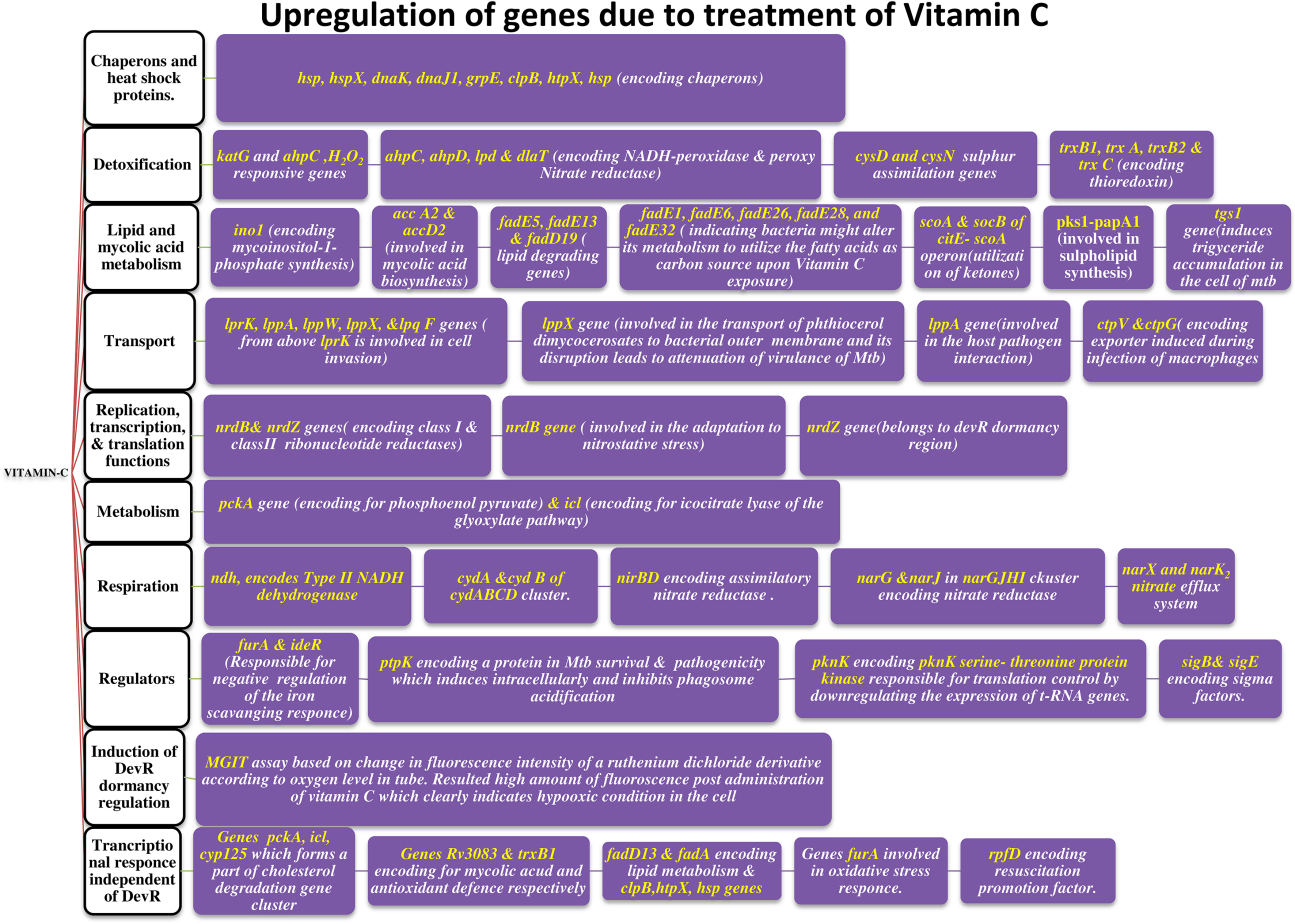
The effect of VC induced Fenton reaction on the bacteria.

Sikri et al., 2015 conducted research on the pleiotropic transcriptional activity of VC in *M. tb.* and found that there were sudden up- and down-regulations of genes involved in the bacterium’s dormancy (**Figure 4**). It was determined that 280 genes, or around 14% of the whole genome, were overexpressed, and that another 14% (283 genes) were silenced. Many categories of these downregulated genes (DRGs) were created based on TubercuList functional classifications. The functional categories “virulence,” “detoxification,” “adaptability,” “lipid metabolism,” “cell wall and processes,” and “intermediary metabolism and respiration” were those with which DRGs were most often associated. 34 (16%) of the 210 genes identified as contributing to “virulence, detoxification, and adaptation” indicated in **Figure 5**, 21 (10%) were noticeably upregulated, whereas3 geneswere significantly downregulated (by up to 4.5-times) (by up to 61-fold). In one crucial area called “lipid metabolism,” 34 genes (or 14% of the total) were upregulated by up to 41-fold and 19 genes (or 8% of the total) were downregulated by up to 3-fold. Yet, of the 736 genes linked to the activities of “cell wall and cell processes,” only 55 (7%) were substantially downregulated when 30 genes (or 4% of the total) were exposed to VC (by up to 4-fold). 81 (or 9%) of the 889 genes on the microarray chip designated as “intermediary metabolism and respiration” were upregulated, while 56 (or 6%) were downregulated, by up to approximately 26-fold and approximately 4-fold, respectively (Sikri et al., 2015).

## 5 Generation of free radicals – *M. tb.* cross-talk with fenton reaction

McCord and Fridovich mentioned the prospect that mammalian cells may create potentially hazardous oxygen-centered free radicals while describing the superoxide dismutase (SOD) family of proteins (as formed by the interaction of ionising radiation with living systems). The job of SOD is evidently to catalyse the dissociation of the superoxide radical (O2.-) and, as a result, to eliminate this species from the system [reaction (I)] (Burkitt and Gilbert, 1990).


(1) $$2O_{2}^{\cdot -}+\;2H^{+}\to H_{2}O_{2}+\;O$$

This species’ apparent cytotoxicity may be due to interactions with redox-active metal ions such as iron and copper, which promote the formation of a more reactive hydroxyl radical. This is related to the fact that 2O_2_.- has a poor sensitivity to biological molecules. The iron-catalyzed Haber-Weiss cycle consists of these iron-catalyzed processes [reactions (2) and (3), where reaction (3) is known as the Fenton reaction] (Burkitt and Gilbert, 1990).,


(2) $$Fe^{3+}+\;O_{2}^{.-}\to Fe^{2+}+O_{2}$$

(3) $$Fe^{3+}+\;H_{2}O_{2}\to Fe^{2+}{+}^{\cdot }OH\;+\;OH^{-}$$

Amaral et al., 2019 showed that necrotic cell death during *M. tb.* infection. Ferroptosis is a type of regulated necrosis induced by accumulation of free iron and toxic lipid peroxides. They observed that Mtb-induced macrophage necrosis is associated with reduced levels of glutathione and glutathione peroxidase-4 (Gpx4), along with increased free iron, mitochondrial superoxide, and lipid peroxidation, all of which are important hallmarks of ferroptosis. Moreover, necrotic cell death in *M. tb.*-infected macrophage cultures was suppressed by ferrostatin-1 (Fer-1), a well-characterized ferroptosis inhibitor, as well as by iron chelation. Additional experiments *in vivo* revealed that pulmonary necrosis in acutely infected mice is associated with reduced Gpx4 expression as well as increasedlipid peroxidation and is likewise suppressed by Fer-1 treatment. Importantly, Fer-1–treated infected animals also exhibited marked reductions in bacterial load. Ferroptosis is initiated by Fenton reaction–induced hydrogen peroxides, which upon interaction with membrane lipids produce toxic lipid peroxides. Under steady state conditions, these lipid peroxides are rapidly reduced by Gpx4 through glutathione (GSH) oxidation. However, when Gpx4 expression and/or activity is inhibited in the presence of excessive iron, lipid peroxide levels become uncontrolled and trigger necrotic cell death. Together, these findings implicate ferroptosis as a major mechanism of necrosis in *M. tb.* infection and as a target for host-directed therapy of tuberculosis **(** Amaral et al., 2019 **)**.

## 6 Important research studies on latent *M. tb.*with vitamin C

One of the most infectious diseases in the world, tuberculosis (TB) infects millions of individuals each year and kills about 40% of them. Due to their capacities for metabolism, pro-oxidation, anti-inflammation, and anti-oxidation, vitamins are essential for many important processes. Treatment for drug-resistant tuberculosis (TB) takes time and is difficult. Although inadequate management of TB treatment can cause the establishment of drug resistance in patients, shortening the length of treatment may significantly improve TB chemotherapy and prevent the development of drug resistance. Inhibition of *M. tb.* growth by VC has been demonstrated. By scavenging oxygen from the culture medium, it induces the dormant state in *M. tb.* It kills the *mycobacteria* by generating reactive oxygen intermediates through iron-mediated Fenton reactions. Many investigations have been done on VC and its effectiveness in killing *M. tb.* (Patti et al., 2021).

Patti. G. et al., investigated the importance of vitamins A, B, C, D, and E in the prevention and treatment of *M. tb.* According to World Health Organization (WHO) recommendations, pyridoxine (Vitamin B6) should be given when TB infection is treated with high-dose isoniazid. According to research, providing vitamin A alone may be more effective in protecting against *M. tb.* Furthermore, it was demonstrated that VC sterilizes drug-susceptible XDR (extremely drug resistant) and MDR (multidrug resistant) cells, as well as prevents the *in vitro* formation of drug-resistant TB. In terms of oxidative equilibrium, vitamin E has shown promise in the treatment of TB (Patti et al., 2021).

Vilcheze et al., described the efficacy of VC against *M. tb.* when combination with first-line drugs rifampicin and isoniazid. A mouse model was employed in this study to examine subinhibitory VC doses in combination with standard first-line medicines. Despite the fact that VC had no effect on *M. tb.*-infected mice, the combination of VC and first-line drugs reduced the bacterial load from the infected mice’s lungs faster than just first-line therapies alone. According to the findings of the study, VC can be used in combination with first-line drugs to treat tuberculosis (Vilchèze et al., 2018).

Taneja et al., looked at how VC affected M. tb’s dormancy, growth arrest, and transcriptional plasticity. Ascorbic acid was shown to cause Dev R regulation by an extra process. Moreover, at around 30% oxygen saturation, ascorbic acid’s oxygen scavenging properties soon trigger Dev R regulation. The magnitude and speed of the responses suggested early Dos T involvement as well as a continual Dev S-mediated response throughout bacterial adaptation to growing hypoxia. Also, it was demonstrated that VC inhibits bacterial growth and promotes the development of the dormancy phenotype in *M. tb.* grown in THP-1 cells in axenic culture (Taneja et al., 2010).

According to study by Shukla et al. published in 2018, the ascorbate (VC) enzyme *M. tb.* ICL (*M. tb.* isocitrate lyase) has the capacity to inhibit M *M. tb.* with an IC50 of 2.15 mM. It is a key enzyme during the latent stage of *M. tb.* No of the amount of iron in the cell, ascorbate at a 4mM concentration stopped 97% of growth. The inhibition produced by 3-nitropropionate, a known inhibitor of *M. tb.* ICL, was also reported to be stronger in the acetate medium than the glucose medium. These findings were corroborated by molecular docking and MD simulation illustrations, which established persistent ascorbate binding to *M. tb.* ICL and enzyme inhibition as a result. Also, it was mentioned that include a high-vitamin diet in a person’s therapy for tuberculosis may have some benefits (Shukla et al., 2018). Peiz et al. studied the pharmacological implications of using VC as a prodrug for the production of hydrogen peroxide, which kills mycobacteria, in 2019. The effectiveness of VC against *M. tb.* H37Rv and *M. bovis* BCG *mycobacteria* in macrophages was examined as part of the study. They examined alterations in protein expression in samples of the H37RV that had been treated with 5mM VC and in control samples using Tandem mass tag (TMT) based quantitative proteomic analysis and qRT-PCR. In their study, they identified 11 genes that were upregulated and 17 genes that were downregulated in 5mM VC-treated H37Rv, including rip 3, fdx A, RV1813c, mtp, LH57 00670, hspX, pfKB, Rv 1824, Rv 1813 c, LH57 08410, and Rv 2030c. Comparison of the H37Rv treated with VC and the control samples. According to qRT-PCR results, VC may express six genes (hsp, fdxD, furA, devR, hspX, and dnaB) in BCG & H37Rv and exons of RAW 264 cells. Seven cells treated with VC killed M. bovis BCG *in vitro*, and it was shown that VC had bactericidal effects on mycobacteria by producing pro-drug hydrogen peroxide (H2O2) and activating the oxidative stress pathway (Pei et al., 2019).

According to Sirkri et al., *M. tb.* exhibits a wide and overlapping pleiotropic transcriptional response to VC. Here, they demonstrated how the pleiotropic effects of VC on various models produced a variety of oxidative stressors. The model was created in a lab, where researchers found that the genes in *M. tb.* cultures that make up around 14% of the genome quickly regulate after being exposed to VC. Genes related in lipid, intermediate metabolism, and regulatory protein were upregulated, whereas genes linked to virulence, detoxification, information flow, and cell wall activities were downregulated. The results showed that VC provided a range of stressful conditions that were comparable to macrophage-like settings for axenic *M. tb.* cells. Moreover, VC causes gaseous stress such as hypoxia, nitric oxide, oxidative, and nitrosative stresses, as well as food restriction. The study found that VC might be utilised to treat *M. tb.* and could prevent the adaptation mechanisms needed for *M. tb.* dormancy (Sikri et al., 2015).

According to studies by Sikri et al., VC becomes more sensitive when administered together with the usage of anti-tuberculosis drugs. According to the study, network-based gene expression analysis revealed that the VC-induced comprehensive and robust adaptive response in *M. tb.*, spanning around 67% of the genome, was brought on by the VC. The bacteria that are resistant to VC show several well-known signs of dormancy, including growth arrest, development of viable but non-culturable cells (VBNC), loss of acid fastness, length reduction, dissipation of reductive stress through glyceride (TAG) accumulation, protective response to oxidative stress, and tolerance to first-line anti-TB medications. The research suggests that pyrazinamide and VC can be combined to provide a strong anti-TB drug with sterilising properties. It also eliminates dormant and reproducing bacteria in both *in vitro* and intracellularly infected mice, independent of any resistance to rifampicin and isoniazid in combination therapy. The findings hint to a successful VC adjuvant therapy that, when combined with already available drugs, boosts efficacy and opens the door to new tactics and combinations (Sikri et al., 2018).

Pal and Jana, 2020 investigated an intracellular Fenton reaction-induced pharmacologic VC-based cell treatment. According to this study, concentrations between 0.1 and 1.0 mM can cause cell death by a Fenton reaction based on iron oxide nanoparticles. The cells can be exposed to VC after being labelled with Fe_3_O_4_ nanoparticles, causing the Fenton reaction to occur following the exposure of VC linked nanoparticles **(** Pal and Jana, 2020 **).**

In 2013, Vilcheze C et al., investigated the sensitivity and lethality of *Mycobacterium tuberculosis* to VC *via* the Fenton reaction. VC, a substance that causes the Fenton reaction, has the ability to kill both drug-susceptible and drug-resistant *Mycobacterium tuberculosis*. Additionally, it claims that VC has a pleiotropic influence on several biological processes and that its ability to combat *Mycobacterium tuberculosis.* is based on high ferrous ion concentrations and the generation of reactive oxygen species **(** Vilchèze et al., 2013 **).**

Tiwari S. et al., investigated how arginine deprivation causes oxidative damage that leads to *M. tb.* sterility. The research demonstrated that the *de novo* arginine biosynthetic pathway of *M. tb.* is upregulated in the early response to the oxidative stress-inducing agent isoniazid or VC deprivation, and that this causes arginine to quickly sterilise the pathway mutants arg B and arg F without the emergence of suppressor mutants *in vitro* or *in vivo*. The results of the transcriptomic cytometry demonstrate a considerable accumulation of ROS and DNA damage. According to metabolomic studies, antioxidant thiol levels were low in cells, and the metabolite substrate for ArgB or ArgF enzymes had accumulated (Tiwari et al., 2018).

In 2015, Mishra A. et al., conducted research on the qualitative and quantitative proteomic analysis of VC-induced changes in *M. smegmatis*. The proteomic analysis revealed significant alterations in cellular and metabolic processes, including reversal of the tricarboxylic acid cycle, reduction of ATP synthase, reduction in iron acquisition and storage, as well as s of the dormancy regulators *WhiB3, PhoP, and Lsr2* **(** Mishra and Sarkar, 2015 **)**.

The effects of vitamin B and VC on DNA methylation and amino acid metabolism in *M. bovis* BCG were examined by Song N et al. The transcriptional, metabolic, and methylation characteristics of *M. bovis* BCG after treatment with vitamin B1 and VC were examined using single molecule real-time sequencing (SMRT), liquid chromatography coupled to mass spectrometry (LCMS), and RNA sequencing (RNA-seq). The results demonstrated that several metabolites were enriched in the metabolic pathways connected to amino acid metabolism. Also, it was established that M4C alterations were only present in *M. bovis* BCG that had undergone treatment, which resulted in an increase of the genes that code for cynteine synthase A. Furthermore, methylation-related genes were upregulated, demonstrating that M4C methylation can marginally boost gene transcription (Song et al., 2020).

The combined effects of anti-tuberculosis medications with VC or N-acetyl cystene (NAC) against bacterial strains of *S. aureus* and *M. tb.*were studied by Khameneh et al., 2016. Here, a 96-well plate was used to test the MIC of every chemical against every strain. Rifampin, a first-line medication, was tested at two-fold concentrations alone, in combination with NAC, or in combination with VC. It was discovered that the MIC of rifampin was reduced to two-fold concentration when treated in combination with VC, whereas NAC had no effect on any drug’s antibacterial activity for all strains of *S. aureus*. However, both VC and NAC exhibit impressive effects on *M. tb.*, and anti-tuberculosis medications. According to the study, VC combined with anti-tuberculosis medications completely eradicates microbiological infections **(** Khameneh et al., 2016 **).**

*Mycobacterium smegmatis*’ ability to build biofilms and maintain long-term life was restricted as a result, Syal et al., 2017. VC that targeted the production of (p)ppGpp. As (p)ppGpp is thought to be the principal regulator of stress response and is in charge of ensuring bacterial survival in stressful situations. The study examined the impact of VC on the production of (p)ppGpp and found that, in contrast to untreated cells, VC-treated *M. smegmatis* cells produced less (p)ppGpp. The findings showed that VC can prevent the manufacture of (p)ppGpp at high doses, and as a consequence, it may be employed as a powerful material to create an inhibitor of (p)ppGpp **(** Syal et al., 2017 **).**

In 2022, Linkon et al. undertook randomised clinical research in tuberculosis patients to compare VC with anti-TB drugs. The research included 32 patients who were randomly assigned to the intervention (VC plus anti-tuberculosis medications) and control (just anti-tuberculosis medications) groups. The control group only received anti-Tuberculosis medications for 28 days whereas the intervention group received 1000 mg of VC daily along with the anti-tuberculosis medications. Sputum smear tests were used for both groups to assess the patients in the second stage. **(** Linkon et al., 2022 **).**

*Citrus aurantifolia* hexane extract’s chemical makeup and some of its compounds’ anti-*M. tb.*,properties were studied by Sandoval-Montemayor NE et al., in 2012. A sensitive and three mono-resistant (isoniazid, streptomycin, or ethambutol) strains of *M. tb.H37Rv* were used in this investigation to identify and characterise the active components from the hexane extract of *Citrus aurantifola* fruit peels. By using column chromatography to separate the active ingredient, five main compounds were produced: 5-methoxypsoralen, 5-geranyloxy-7-methoxycoumarin, 5,7-dimethoxycoumarin, and 5,8-dimethoxypsoralen. NMR spectroscopy in the 1D and 2D ranges was used to describe the structure. Additionally, the extract underwent GC-MS analysis, and many chemicals were identified. Using the microplate alamar blue assay, three multidrug resistant *M. tb.* strains and further *M. tb. H37Rv* were tested against four isolated coumarins and 16 commercial chemicals identified by GC-MS. The constituents 5,8-dimethoxypsoralen, 5-geranyloxypsoralen, palmitic acid, linoleic acid, oleic acid, 4-hexen-3-one, and citrus shown efficacy against all strains. According to the study, the hexane extract of *C. aurantifolia* shows anti - mycobacterial properties **(** Sandoval-Montemayor et al., 2012 **).**

In 2016, Kirtania et al., investigated the effects of VC-driven *Mycobacterium smegmatis* growth on Dev R-dependent synchronization and mycobacteriophage *D29* proliferation. It was demonstrated using *Mycobacterium smegmatis* - mycobacteriophage *D29* as a model system to examine how the growth of mycobacteriophages is influenced by hypoxic conditions brought on by VC that previous exposure of the host to such conditions resulted in larger burst size of the phage. VC pre-exposure was also shown to promote simultaneous growth in the host. The two occurrences are linked as a consequence. The response regulator *DevR*, which controls mycobacteria’s hypoxic responses, is absent in a mutant that cannot tolerate larger phage bursts or undergo synchronized growth in response to VC pre-exposure. This link was further supported by the discovery that phage burst sizes varied according to the stage of synchronous growth that the host cells were in at the time of infection. Lower bursts were seen during the dividing phases and higher ones during the resting/synthetic phases. The effects were specific in nature since synchronizationutilizing the unrelated method of “crowding” did not generate the same outcome. The results show that mycobacteriophage D29 uses VC-induced growth synchronization, a DevR-dependent phenomenon, to replicate **(** Kirtania et al., 2016 **).**

## 7 Discussion

Drug-resistant TB is spreading globally, necessitating the creation of novel, potent treatments, as well as innovative approaches to combat it. The problem has become more complicated as a result of resistance to recently authorised anti-tuberculous medications. In this case, the role of vitamins is being reevaluated. Vitamins are more readily accepted by doctors caring for patients due to their proven efficacy in improving health and a general sense of well-being. In the past, there have been several attempts to treat TB with dietary supplements that include different vitamins (Tyagi et al., 2017).

The effect of VC on various ailments has been thoroughly explored but remains debatable. VC is widely known for being both an antioxidant and a pro-oxidant. VC’s pro-oxidant action is most likely what causes it to sterilizes *M. tb.* cultures. VC generates ROS, produces redox imbalance, and damages DNA, all of which result in antibacterial capabilities against *M. tb.* cells. When fed to *M. tb.* in an environment with low oxygen or iron, VC loses its antibacterial activity (Vilchèze et al., 2013). It may even inhibit the bacterial katG’s ability to neutralize ROS. By suppressing ICL, ascorbate, on the other hand, may hinder bacteria from metabolizing fatty acids in fatty acid-rich and iron-depleted microenvironments (Shukla et al., 2018).

The study’s goal was to determine the drugs used to treat mycobacterial infections, as well as the mechanism of drug resistance and the impact of VC/first-line therapies for MDR and XDR infections. The authors expect that the findings of this review will help them better understand the probable underlying mechanism in the link between resistant tuberculosis and vitamin C, its pro-oxidant activity (free radical formation), and first-line treatments.

## Author contributions

RSK and PG designed and drafted the MS. RSK and TKU reviewed and edited the MS. RSK and MD revised the MS. IA and MS contributed in revision, reviewing and English editing, grammatical corrections and funding acquisition.
